# Fructose Reduces Mitochondrial Metabolism and Increases Extracellular BCAA during Insulin Resistance in C2C12 Myotubes

**DOI:** 10.3390/nu16111582

**Published:** 2024-05-23

**Authors:** Norah E. Cook, Macey R. McGovern, Toheed Zaman, Pamela M. Lundin, Roger A. Vaughan

**Affiliations:** 1 Department of Health and Human Performance, High Point University, One University Parkway, High Point, NC 27268, USA; ncook@highpoint.edu (N.E.C.); mmcgove4@highpoint.edu (M.R.M.); 2 Department of Chemistry, High Point University, High Point, NC 27268, USA; tzaman@highpoint.edu (T.Z.); plundin@highpoint.edu (P.M.L.)

**Keywords:** leucine, isoleucine, valine, skeletal muscle, lipogenesis, insulin sensitivity

## Abstract

Fructose is a commonly consumed monosaccharide implicated in developing several metabolic diseases. Previously, elevated branched-chain amino acids (BCAA) have been correlated with the severity of insulin resistance. Most recently, the effect of fructose consumption on the downregulation of BCAA catabolic enzymes was observed. Thus, this mechanistic study investigated the effects of physiologically attainable levels of fructose, both with and without concurrent insulin resistance, in a myotube model of skeletal muscle. Methods: C2C12 mouse myoblasts were treated with fructose at a concentration of 100 µM (which approximates physiologically attainable concentrations in peripheral circulation) both with and without hyperinsulinemic-mediated insulin resistance. Gene expression was assessed by qRT-PCR, and protein expression was assessed by Western blot. Oxygen consumption rate and extracellular acidification rate were used to assess mitochondrial oxidative and glycolytic metabolism, respectively. Liquid chromatography-mass spectrometry was utilized to analyze leucine, isoleucine and valine concentration values. Results: Fructose significantly reduced peak glycolytic and peak mitochondrial metabolism without altering related gene or protein expression. Similarly, no effect of fructose on BCAA catabolic enzymes was observed; however, fructose treatment resulted in elevated total extracellular BCAA in insulin-resistant cells. Discussion: Collectively, these observations demonstrate that fructose at physiologically attainable levels does not appear to alter insulin sensitivity or BCAA catabolic potential in cultured myotubes. However, fructose may depress peak cell metabolism and BCAA utilization during insulin resistance.

## 1. Introduction

Fructose is a monosaccharide that shares many similarities with glucose, including empirical formula and several aspects of metabolism; however, several important differences in absorption and metabolism between glucose and fructose have been well-documented. The study of these basic biochemical properties across various tissues, including the comprehensive study of fructose metabolism via the glycolytic pathway, has identified that fructose may uniquely contribute to several metabolic conditions by enhanced lipogenesis [1,2,3], especially when consumed in excess along with other lifestyle factors such as sedentarism [4]. As a result, excess fructose may lead to enhanced lipogenesis and diminished mitochondrial fatty acid oxidation, thereby inhibiting mitochondrial function [1]. Additionally, fructose has been shown to increase de novo lipogenesis in the liver, which promotes lipid accumulation in various tissues. The resultant accumulation of excess lipids promotes insulin resistance and other metabolic consequences. Fructose is thought to act in part by activation of carbohydrate response element binding protein (ChREBP) as well as peroxisome proliferator-activated receptor gamma (PPARγ), both of which contribute to the biosynthesis of lipids for storage. Interestingly, activation of ChREBP has also been shown to downregulate other metabolic processes, such as the metabolism of branched-chain amino acids (BCAA) [5,6].

BCAA accumulation during metabolic disease has been observed in a variety of settings and populations [7,8,9,10,11,12,13,14,15], leading some to speculate that targeting BCAA metabolism may provide a therapeutic option for treating or preventing insulin resistance. Though the exact mechanisms responsible for the accumulation of BCAA during insulin resistance are still debated to some extent, the downregulation of BCAA catabolic enzymes is thought to be a primary contributing factor. While substantially lower in concentration than other carbohydrates in the peripheral circulation (namely glucose), elevated fructose levels have also been reported in populations with insulin resistance [16]. Given the potential correlation between fructose and BCAA levels, some have investigated the potential causal relationship between the two. Specifically, fructose appears to downregulate the activation of the rate-limiting enzyme in the catabolism of BCAA, branched-chain alpha-keto acid dehydrogenase (BCKDH) by modifying the abundance of the regulatory kinase (BCKDK) and phosphatase (PPM1K) of BCKDH [5]. While some of the effects of fructose on BCAA metabolism have been explored, the effects of fructose on muscle metabolism are less understood. Because skeletal muscle represents the primary site of BCAA metabolism and is also indispensable for insulin sensitivity, understanding the effects of fructose on muscle metabolism is important.

Most recently, David et al. investigated the effects of chronic fructose consumption in rats and found time-dependent effects on glucose homeostasis, as well as increased circulating BCAA following 45 days of high-fructose feeding [17]. The report further investigated the effects of fructose feeding on the expressional profiles of BCAA catabolic enzyme expression in various tissues and found reduced BCAT2 expression in skeletal muscle, but not liver or either subcutaneous or epididymal adipose. Similarly, David et al. showed increased pBCKDHA expression in skeletal muscle (indicative of reduced BCAA catabolism) but not liver or adipose tissues. The report also revealed that fructose reduced mRNA expression of key regulators of mitochondrial function/content and carbohydrate-mediated lipogenesis, specifically peroxisome proliferator-activated receptor gamma coactivator 1-alpha (Ppargc1a) and ChREBP as MLX interacting protein-like (Mlxipl) mRNA, respectively [17]. Similar observations were made by Yu et al., who showed that rats given a high-fructose diet for 8 weeks displayed increased circulating BCAA and reduced Bckdha mRNA expression and BCKDA immunofluorescence in skeletal muscle [18]. One important question that arises from the observations by David et al. is whether fructose is directly responsible for the downregulation of BCAA catabolic capacity within skeletal muscle or if the effects are secondary to insulin resistance, which is known to be associated with reduced BCAA catabolic capacity. To investigate the direct effects of fructose on BCAA catabolic capacity as well as the general metabolic phenotype, we used cultured myotubes treated with and without physiologically attainable levels of fructose, both with and without concurrent insulin resistance.

Metabolically, the effect of excess fructose in myotubes was found fructose upregulated metabolic regulators such as AMPK mRNA and protein activation (pAMPK), as well as increased mRNA of peroxisome proliferator-activated receptor alpha (Ppara) [19]. Another study assessed the effect of 15 mM fructose on antioxidant capacity and mitochondrial function in L6 myotubes and showed that fructose reduced mitochondrial function following 48 but not 24 h [20]. Similarly, AMPK phosphorylation was lower after 48 h of treatment but not at earlier time points. Interestingly, the report also showed reduced ATP production as early as 3 h, which was also observed in the assessment of mitochondrial complex function, suggesting dysregulation of ATP production may precede alterations in in-tact mitochondrial function [20]. Not surprisingly, the report also showed increased apoptosis, though importantly, these experiments were performed at exceedingly high levels of fructose beyond what is achievable in the blood [20]. Thus, in vitro data demonstrates a negative effect of fructose on muscle metabolism; however, an important limitation of these reports was the use of 15–25 mM fructose, which is not physiologically attainable. In fact, circulating levels of fructose in peripheral circulation tend to be substantially lower than glucose. In rats, fructose disproportionally increased lipid content and glucose intolerance to a greater extent in adult versus young animals [21]. Consistent with other observations, fructose reduced AMPK activation as well as Sirt1 expression yet resulted in a perplexing increase of PGC-1 mitofusin 2 and DRP1 in skeletal muscle [21]. Despite these alterations, no changes in skeletal muscle protein expression of mitochondrial respiratory chain components or cytochrome c oxidase activity were observed.

Collectively, data demonstrates high fructose consumption may be associated with metabolic pathology due to its high lipogenic potential [1,2,3,4]. Additionally, emerging data suggest that despite the dramatically lower levels, circulating fructose is still a correlate and predictor of insulin resistance [16]. Thus, although fructose has been linked with altered metabolism and has been implicated both in the development of insulin resistance and the loss of BCAA catabolic capacity, experimental data have yet to fully elucidate the full metabolic effects of fructose at physiologically attainable levels. The purpose of the present report was to assess the effects of the highest physiologically attainable levels of peripherally circulating fructose as seen during insulin resistance on various aspects of myotube metabolism. To our knowledge, this is the first report to investigate the effects of physiologically attainable levels of fructose as seen during insulin resistance, both with and without concurrent insulin resistance, in a myotube model of skeletal muscle.

## 2. Materials and Methods

### 2.1. Cell Culture

C2C12 mouse myoblasts from ATCC (Manassas, VA, USA) were cultured in Dulbecco’s Modified Eagle’s Medium (DMEM) containing 4500 mg/L glucose and 20% heat-inactivated fetal bovine serum (FBS) and 100 U/mL penicillin and 100µg/mL streptomycin in a humidified 5% CO_2_ atmosphere at 37 °C. Cells were grown to confluency with growth media changed every two to three days (using cell passages <20 for all experiments). Differentiation was accomplished by replacing growth media with DMEM with 2% horse serum, 100 U/mL penicillin, and 100 µg/mL streptomycin for 6–9 days. Similar to previous experiments [22,23,24,25,26], insulin resistance was accomplished by adding insulin at 100 nM for the final 3 days of differentiation, which significantly reduces insulin signaling without altering cell differentiation status [25,26]. D-fructose from Sigma was dissolved in sterile water, and cells were treated with differentiation media with fructose at a final concentration of 100 µM (0.1% water vol:vol) or sterile water control (0.1% water vol:vol) for 24 h, both with and without concurrent insulin resistance. This concentration of fructose was chosen because subjects with insulin resistance show elevated systemic fructose levels, the highest of which is approximately 100 µM [16]. Similar observations have been made in rodents fed a high-fructose diet [27]. Because 100 µM approximates the highest level of fructose observed in the peripheral circulation in insulin-resistant populations [16], we assumed that meaningful alterations in cell metabolism and related outcomes would be measurable following 24 h of treatment at this level. Thus, given the purpose of this report is to assess the effect of physiologically attainable levels of fructose on muscle metabolism, these systemic levels appear to be the highest achievable via dietary practices and an appropriate level for these experiments.

### 2.2. Quantitative Real-Time Polymerase Chain Reaction (qRT-PCR)

Following treatment, total mRNA was extracted using the Trizol method and quantified (via NanoDrop from Thermo Fisher, Wilmington, DE, USA), and cDNA was synthesized using the iScript cDNA Synthesis Kit from Bio-Rad (Hercules, CA, USA) according to manufacturer’s instructions. PCR primers were synthesized by Integrated DNA Technologies (Coralville, IA, USA) (Table S1). Amplification of target genes was normalized to the housekeeping gene, Tata binding protein, which did not differ between groups (Tbp, shown in Figure S1). qRT-PCR reactions were performed using the CFX Connect System from Bio-Rad (Hercules, CA, USA). SYBR Green-based PCR was performed using final primer concentrations at 3.75 µM in a total volume of 10 µL per well. The following cycling parameters were used: 95 °C for 3 min followed by 40 cycles of 95 °C for 15 s, and 60 °C for 30 s. qRT-PCR reactions were performed using *n* = 3 per treatment condition from two independent experiments with *n* = 6 for the final analysis. Relative quantification was determined via the ΔΔCt method.

### 2.3. Immunoblotting

Cells were differentiated and treated as described above, followed by serum-free media stimulation with 100 nM insulin for 30 min. Whole-cell lysates were then prepared by harvesting the cells on ice in RIPA buffer supplemented with protease inhibitor, followed by incubation on ice for 60 min. Insoluble material was removed, and protein concentrations were determined using the Bradford assay. Total protein (50 μg per sample) was size-separated by 10% sodium dodecyl sulfate-polyacrylamide gel electrophoresis (SDS-PAGE) and electro-transferred to PVDF membranes. After blocking in TBST-5% non-fat milk powder for 1 h, membranes were probed at 4 °C overnight with primary antibodies in TBST-5% non-fat milk powder (details in Table S2). Bound antibodies were detected by horseradish peroxidase-conjugated secondary antibodies from Abcam (Cambridge, MA, USA) at a dilution of 1:5000 in TBST-5% non-fat milk powder for 1 h at room temperature while shaking (details in Table S2). Protein signal intensities were determined by chemiluminescence using the Clarity Western ECL substrate kit from Bio-Rad (Hercules, CA, USA) and imaged using the ChemiDoc Touch from Bio-Rad (Hercules, CA, USA). Relative signal intensities were quantified using Image Lab from Bio-Rad (Hercules, CA, USA). Blots were performed using three replicates per condition performed across two independent cell culture experiments with *n* = 6 for the final analysis. Molecular weights for all targets were verified against sizes suggested by product brochures. Importantly, loading controls did not differ between groups (Figure S2).

### 2.4. Seahorse Metabolic Assays

Cells were seeded into Seahorse XFe96 culture plates, differentiated, and treated as described above. Media was then replaced with XF Assay Media obtained from Agilent Technologies (Santa Clara, CA, USA) containing glucose at 25 mM, pyruvate at 1 mM, and glutamine at 2 mM. Following incubation, baseline measurements of oxygen consumption rate (OCR) and extracellular acidification rate (ECAR) were recorded as indicators of basal oxidative metabolism and glycolytic metabolism, respectively. Following basal measurements, each well was infused with oligomycin (an inhibitor of ATP synthase) at a final concentration of 2 μM to induce maximal glycolytic metabolism. Cells were then exposed to carbonyl cyanide p-[trifluoromethoxy]-phenyl-hydrazone (FCCP) at 2 μM to uncouple electron transport and induce peak OCR. Maximal respiration measurements were followed by the injection of rotenone at 1 μM to reveal non-mitochondrial respiration. Basal and peak oxidative metabolism were normalized to non-mitochondrial OCR from each respective well. The Seahorse XFe96 Analyzer was run using a 6 min cyclic protocol command (mix for 3 min and measure for 3 min). MitoStress assays included *n* = 23 per group, repeated with two independent experiments for *n* = 46 per group for the final analysis. States of mitochondrial metabolism were calculated by subtracting non-mitochondrial respiration from basal, oligomycin-mediated proton leak, or FCCP-induced peak mitochondrial oxygen consumption. No wells showed negative OCR values or lack of response to injection.

### 2.5. Fluorescent Staining and Microscopy

Immediately following the Seahorse metabolic assay described above, cells were fixed using 3.7% formaldehyde at 37 °C with a 5% CO_2_ atmosphere. The fixing agent was removed, cells were stained with DAPI at 0.5 µM in PBS, and fluorescence was measured at 360/460 nm (Figure S3). Cells were then stained with 100 µM nonyl acridine orange (NAO) (Fremont, CA, USA) in PBS and incubated in the dark at room temperature for 10 min. Fluorescence was then measured using 485/525 nm excitation/emission to reveal mitochondrial staining. Neutral lipid content was measured using Nile Red staining at 10 µM PBS with 1% DMSO vol/vol using 530/645 nm excitation/emission. All fluorescent measurements were made in triplicate, and the average (less background) analyzed with *n* = 23 per group was repeated with two independent experiments with *n* = 46 per group for the final analyses. Following fluorescent quantification, cells were imaged using the 20X objective using the Motic AE31E inverted microscope and Moticam Pro 252B (Causeway Bay, Hong Kong, China).

### 2.6. Liquid Chromatography–Mass Spectrometry (LC–MS)

Chromatographic separation and quantification of leucine, isoleucine, and valine was performed using a Shimadzu Nexera UHPLC system equipped with a Phenomenex Kinetex C18 100Å column (100 × 3 mm, 2.6 µm) kept at a temperature of 30 °C connected to Shimadzu LCMS-8045 triple quadrupole mass spectrometer (Shimadzu, Kyoto, Japan) fitted with a DUIS ion source [28]. The source used nebulizer gas of 2.0 L/min, drying gas of 10.0 L/min, desolvation line (DL) temperature of 250 °C and heat block temperature of 400 °C, with CID gas 230 kPa. The mobile phases of A (water with 0.1% formic acid) and B (methanol 0.1% formic acid) were used at a flow rate of 0.4 mL/min for the following gradient method: 0 min, 20% B; 1.7 min, 40% B; 5.0 min, 65% B; 8.0 min, 65% B; followed by 4 min 20% B for column equilibration. The injection volume was maintained at 1 µL. This afforded reproducible retention time values for valine (1.589 min), isoleucine (2.093 min), and leucine (2.213 min).

Shimadzu LabSolution software version 5.97 was used to acquire and process the data. The fragmentation for each BCAA was optimized using MRM set to positive mode for valine (118.1 to 72.2 m/z, Q1 −23.0 V, CE −12.0 V, and Q3 −20.0 V), isoleucine (132.0 to 69.2 m/z, Q1 −10.0 V, CE −19.0 V, and Q3 −11.0 V), and leucine (132.1 to 43.2 m/z, Q1 −10.0 V, CE −26.0 V, and Q3 −18.0 V), with a dwell time of 100 ms.

A stock solution containing all BCAAs at a concentration of 8.0 mM was obtained by dissolving each amino acid in water/methanol solution (50:50, *v*/*v*) and kept at 4 °C. Further dilutions with water/methanol were performed to assemble a calibration curve ranging from 3.125 to 100.0 µM. Experiments were performed using three replicates per group for each of two independent cell culture experiments with *n* = 6 for each group in the final analyses.

### 2.7. Statistical Analyses

Data are presented as dot plots with group means or as group mean ± SE. Data were analyzed with two-way ANOVA followed by subsequent one-way ANOVA in order to assess between-group differences. Bonferroni’s correction was used for all group comparisons. Values of *p* < 0.05 were used to identify significant differences between groups.

## 3. Results

### 3.1. Effect of Physiological Fructose on Myotube Insulin Sensitivity

**Figure 1:**
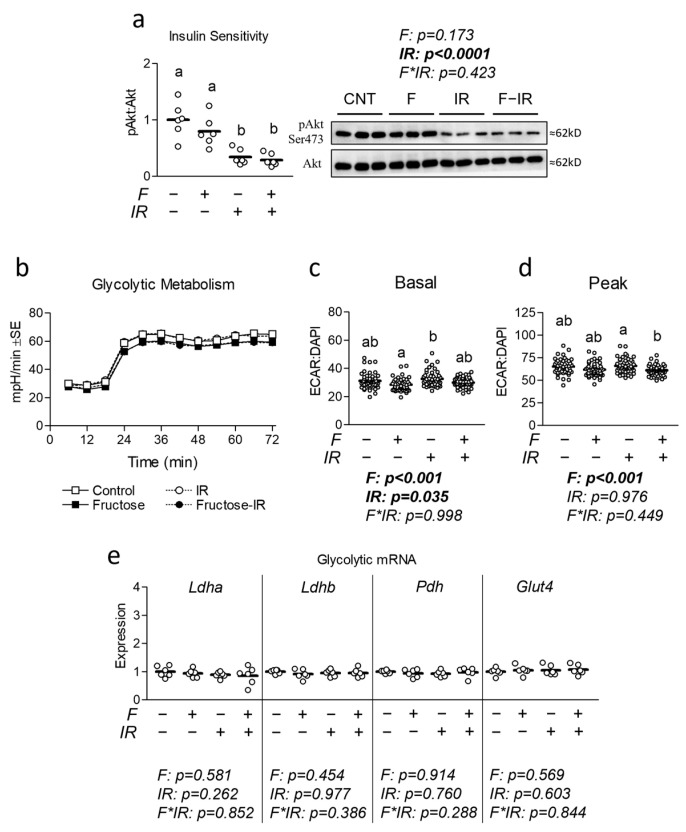
Effect of fructose on myotube insulin sensitivity and glycolytic metabolism. (**a**) Effect of fructose at 100 µM for 24 h with and without insulin resistance (IR) on insulin sensitivity following 30 min insulin stimulation at 100 nM. (**b**) Time course of the effect of fructose at 100 µM for 24 h with and without IR on glycolytic metabolism. (**c**,**d**) Effect of fructose with and without IR on (**c**) basal and (**d**) peak glycolytic metabolism normalized to cell nuclei content (presented in Figure S3). (**e**) Effect of fructose with and without IR on mRNA expression of lactate dehydrogenase a (Ldha), lactate dehydrogenase b (Ldhb), pyruvate dehydrogenase (Pdh), and Solute Carrier Family 2 Member 4 (Slc2a4/Glut4). Notes: Data were analyzed using two-way ANOVA followed by one-way ANOVA with Bonferroni’s correction for multiple comparisons used to assess differences in metabolism, gene expression, and insulin signaling. Groups with dissimilar letters indicate *p* < 0.05 between groups. Metabolic measurements were performed using *n* = 23 individual replicates per treatment condition and were repeated across two independent cell culture experiments with *n* = 46 per group in the final analyses. No wells responded with negative raw values. Target gene expression was normalized to average Tata binding protein (Tbp) using three replicates per group across two independent cell culture experiments with *n* = 6 for the final analysis. Western blots were performed using three replicates per group across two independent cell culture experiments with *n* = 6 for the final analysis and were normalized to total Akt.

To investigate the effects of physiologically attainable levels of fructose as seen during insulin resistance, both with and without concurrent insulin resistance on myotube insulin sensitivity, we began by assessing the effect of fructose on insulin signaling in both insulin-sensitive and insulin-resistant cells (Figure 1). As expected, insulin-resistant cells exhibited a significantly lower response in pAkt activation (a central component of insulin signaling [29]) following insulin stimulation, while no effect of fructose was observed (Figure 1a). Because excess fructose has been associated with reduced insulin sensitivity, we examined the effect of fructose with and without insulin resistance on glycolytic metabolism (Figure 1b). Significant main effects for both fructose and insulin resistance were observed in basal glycolytic metabolism, suggesting both treatment conditions contribute to reduced glycolytic metabolism (Figure 1c). Interestingly, only fructose treatment showed a significant main treatment effect for reduced peak glycolytic metabolism (Figure 1d). Because fructose treatment reduced glycolytic metabolism, we measured the effect of fructose, with and without IR, on mRNA expression of targets associated with glucose metabolism including lactate dehydrogenase a (Ldha), lactate dehydrogenase b (Ldhb), pyruvate dehydrogenase (Pdh), and Solute Carrier Family 2 Member 4 (Slc2a4/Glut4); however, no significant alterations were observed across any of the measured targets (Figure 1e). 

### 3.2. Effect of Physiological Fructose on Myotube Mitochondrial Metabolism and Content

**Figure 2:**
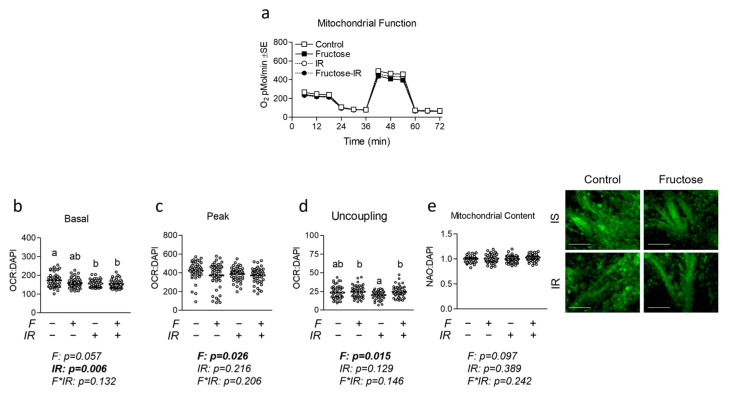
Effect of fructose both with and without insulin resistance on mitochondrial metabolism and content. (**a**) Time course of the effect of fructose at 100 µM for 24 h with and without insulin resistance (IR) on mitochondrial metabolism. (**b**,**c**) Effect of fructose with and without IR on (**b**) basal, (**c**) peak, or (**d**) proton leak mitochondrial metabolism normalized to cell nuclei content (presented in Figure S3a). (**e**) Mitochondrial content of cells described in “a” indicated by NAO staining following normalization to cell nuclei content (presented in Figure S3a). Notes: Data were analyzed using two-way ANOVA followed by one-way ANOVA with Bonferroni’s correction for multiple comparisons used to assess differences in metabolism, mitochondrial content, and gene expression. Groups with dissimilar letters indicate *p* < 0.05 between groups. Metabolic measurements were performed using *n* = 23 individual replicates per treatment condition and were repeated across two independent cell culture experiments with *n* = 46 per group in the final analyses. No wells responded with negative raw values. Images in panel “e” were obtained using a 20× objective with the white scale bar indicating 150 µm.

**Figure 3:**
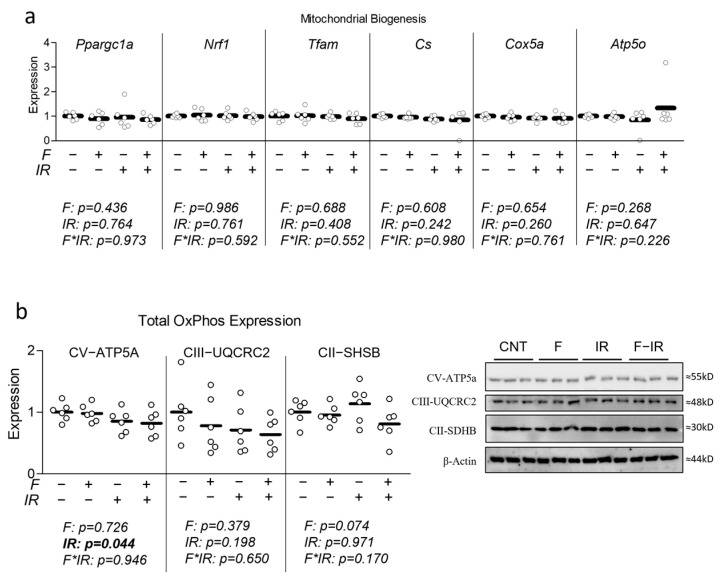
Effect of fructose both with and without insulin resistance on mitochondrial biogenesis and respiratory component expression. (**a**) Effect of fructose at 100 µM for 24 h with and without insulin resistance (IR) on mRNA expression of mitochondrial biogenesis including peroxisome proliferator-activated receptor-gamma coactivator-1alpha (Ppargc1a), nuclear respiratory factor 1 (Nrf1), mitochondrial transcription factor A (Tfam), citrate synthase (Cs), cytochrome c oxidase subunit 5a (Cox5a), and ATP synthase F0 (Atp5o). (**b**) Effect of fructose with and without IR on protein expression of mitochondrial respiratory proteins, including ATP synthase (ATP5A), complex 3 (CIII-UQCRC2), and complex 2 (CII-SDHB). Notes: Data were analyzed using two-way ANOVA followed by one-way ANOVA with Bonferroni’s correction for multiple comparisons used to assess differences in gene and protein expression. No group differences were observed. Target gene expression was normalized to average Tata binding protein (Tbp) using three replicates per group across two independent cell culture experiments with *n* = 6 for the final analysis. Western blots were performed using three replicates per group across two independent cell culture experiments with *n* = 6 for the final analysis and were normalized to β-actin.

Next, to assess the effects of physiologically attainable levels of fructose on myotube metabolism, we examined the effects of fructose on mitochondrial function, content, and related gene expression. For mitochondrial function (Figure 2a), both insulin-resistant groups (with and without fructose) displayed significantly lower basal mitochondrial respiration than insulin-sensitive cells (Figure 2b). However, fructose reduced peak mitochondrial function, as indicated by a significant main effect of fructose (Figure 2c), though no group differences were observed. Conversely, fructose increased mitochondrial proton leak in both insulin-sensitive and resistant cells, suggesting altered mitochondrial respiratory efficiency (Figure 2d). We also assessed mitochondrial content via mitochondrial staining; however, neither fructose nor insulin resistance altered the mitochondrial content (Figure 2e). To verify the lack of alteration of mitochondrial content, we measured gene expression of targets associated with the regulation of mitochondrial content/function including nuclear respiratory factor 1 (Nrf1), mitochondrial transcription factor A (Tfam), citrate synthase (Cs), cytochrome c oxidase subunit 5a (Cox5a), and ATP synthase (Atp5o). We again found no significant differences between any of the tested groups (Figure 3a). We further assessed the expression of mitochondrial respiratory components at the protein level and found a significant main effect of insulin resistance for suppressing ATP5A expression, but no significant group differences were observed (Figure 3b). No significant differences were observed in other respiratory proteins (Figure 3b).

### 3.3. Effect of Physiological Fructose on Myotube Lipid Content and Lipogenesis

**Figure 4:**
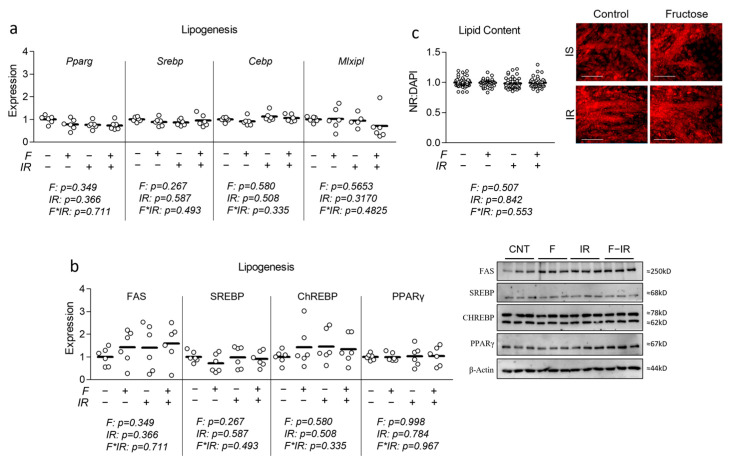
Effect of fructose both with and without insulin resistance on lipid content and lipogenic signaling. (**a**) Effect of fructose at 100 µM with and without insulin resistance (IR) on mRNA expression of lipogenesis including peroxisome proliferator-activated receptor-gamma (Pparg), sterol response element binding protein (Srebp), CCAAT/enhancer-binding protein alpha (Cebpa), and carbohydrate response element binding protein as MLX interacting protein-like (Mlxipl). (**b**) Effect of fructose with and without IR on protein expression of fatty acid synthase (FAS), sterol response element binding protein (SREBP), Carbohydrate response element binding protein (ChREBP), and peroxisome proliferator-activated receptor-gamma (PPARγ). (**c**) Effect of fructose with and without IR on lipid content indicated by Nile Red staining following normalization to cell nuclei content (presented in Figure S3a). Notes: Data were analyzed using two-way ANOVA followed by one-way ANOVA with Bonferroni’s correction for multiple comparisons used to assess differences in gene expression, protein expression, and lipid content. Groups with dissimilar letters indicate *p* < 0.05 between groups. Lipid content measurements were performed using *n* = 23 individual replicates per treatment condition and were repeated across two independent cell culture experiments with *n* = 46 per group in the final analyses. Target gene expression was normalized to average Tata binding protein (Tbp) using three replicates per group across two independent cell culture experiments with *n* = 6 for the final analysis. Western blots were performed using three replicates per group across two independent cell culture experiments with *n* = 6 for the final analysis and were normalized to β-actin. Images in panel (**c**) were obtained using a 20× objective with the white scale bar indicating 150 µm.

Fructose has previously been linked with increased lipogenic signaling and lipid content. Thus, we began our investigation by assessing the effect of physiological levels of fructose on lipogenic signaling. Similar to mRNA expression of genes associated with either glycolytic metabolism and mitochondrial biogenesis/function, neither fructose nor insulin resistance altered expression of lipogenic regulators including proliferator-activated receptor-gamma (Pparg), sterol response element binding protein (Srebp), CCAAT/enhancer-binding protein alpha (Cebpa), or Mlxipl (mRNA transcript of ChREBP) (Figure 4a). Because fructose is known to induce lipogenesis in various tissues, we also assessed the expression of lipogenic targets at the protein level and found no significant effect of either treatment condition on fatty acid synthase (FAS), sterol response element binding protein (SREBP), ChREBP, or PPARy (Figure 4b). To verify the lack of change in gene and protein expression at the lipid content level, cells were stained with Nile Red following treatment, and again, no effect of either treatment on total lipid content was observed (Figure 4c). 

### 3.4. Effect of Physiological Fructose on Myotube BCAA Cataolic Enzyme and Extracellular BCAA

**Figure 5:**
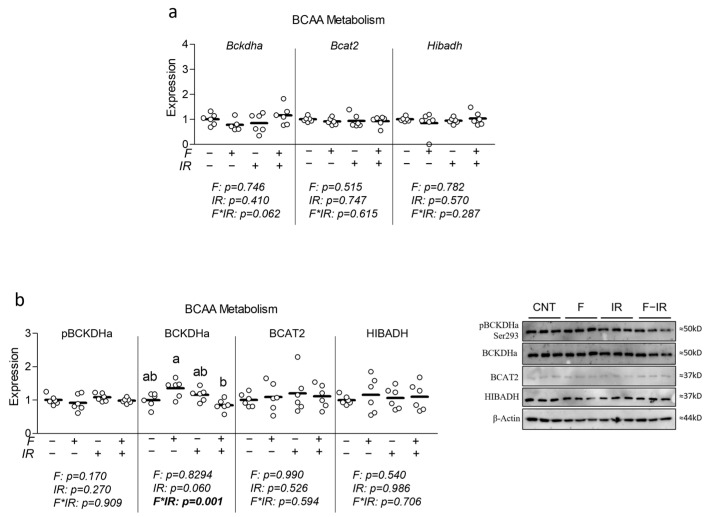
Effect of fructose with and without insulin resistance on myotube BCAA metabolism. (**a**) Effect of fructose at 100 µM for 24 h with and without insulin resistance (IR) on mRNA expression of branched-chain alpha-keto acid dehydrogenase (Bckdha), branched-chain aminotransferase 2 (Bcat2), and hydroxyisobutyrate dehydrogenase (Hibadh). (**b**) Effect of fructose with and without IR on protein expression of phospho-branched-chain alpha-keto acid dehydrogenase (pBCKDHa), branched-chain alpha-keto acid dehydrogenase (BCKDHa), branched-chain aminotransferase 2 (BCAT2), and hydroxyisobutyrate dehydrogenase (HIBADH). Notes: Data were analyzed using two-way ANOVA followed by one-way ANOVA with Bonferroni’s correction for multiple comparisons used to assess differences in gene expression and protein expression. Groups with dissimilar letters indicate *p* < 0.05 between groups. Target gene expression was normalized to average Tata binding protein (Tbp) using three replicates per group across two independent cell culture experiments with *n* = 6 for the final analysis. Western blots were performed using three replicates per group across two independent cell culture experiments with *n* = 6 for the final analysis and were normalized to either total BCKDHA (in the case of pBCKDHa) or β-actin (all other targets).

**Figure 6:**
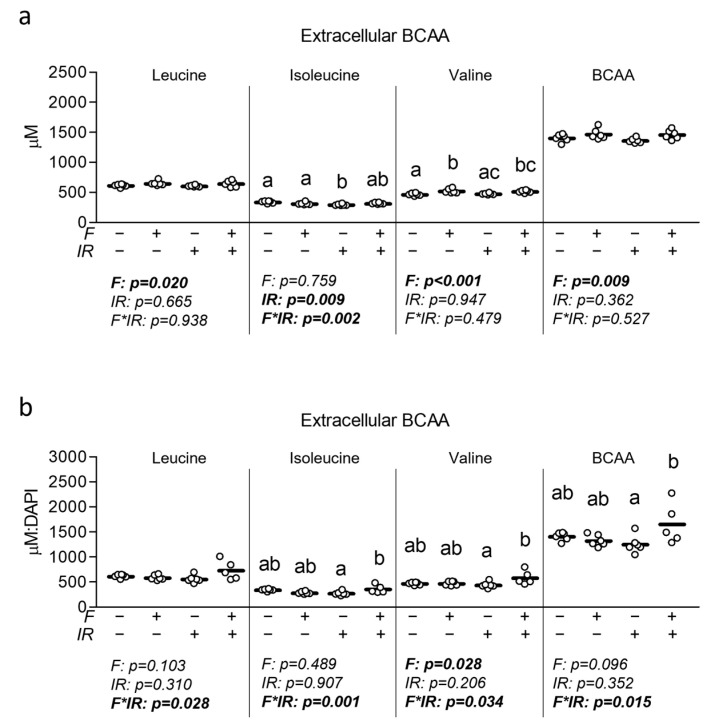
Effect of fructose with and without insulin resistance on extracellular myotube BCAA accumulation. (**a**) Effect of fructose at 100 µM for 24 h with and without insulin resistance (IR) on extracellular leucine, isoleucine, valine, or cumulative BCAA. (**b**) Effect of fructose with and without IR as described in “a” following normalization to relative nuclei content (Figure S3b). Notes: Data were analyzed using two-way ANOVA followed by one-way ANOVA with Bonferroni’s correction for multiple. Groups with dissimilar letters indicate *p* < 0.05 between groups. Each metabolite was assessed using three replicates per group across two independent cell culture experiments with *n* = 5–6 for the final analysis, with each sample measured in technical triplicate (one nuclei-normalized value was removed as an outlier for the fructose with insulin resistance group).

Lastly, because David et al. provided direct observations that fructose consumption may result in the down-regulation of skeletal muscle BCAA catabolic capacity [17], we assessed the effect of a physiologically high but attainable level of fructose with and without insulin resistance on mRNA and protein expression of BCAA catabolic enzymes. We first assessed the effect of fructose with and without IR on mRNA expression of Bckdha, Bcat2, and hydroxyisobutyrate dehydrogenase (Hibadh) but again observed no significant effect of either treatment on BCAA catabolic gene expression (Figure 5a). We also assessed each of these BCAA catabolic enzymes at the protein level. While we did not observe any changes in the relative activity of BCKDHa (defined by phosphorylation at Ser293), we observed a significant interaction effect for total BCKDHa expression during which cells that were insulin-resistant and co-treated with fructose displayed reduced total BCKDHa expression versus fructose-only treated cells (Figure 5b). However, no significant differences were observed in BCAT2 or HIBADH at the protein level (Figure 5b). We further explored the effect of fructose with and without concurrent insulin resistance on extracellular BCAA content and found significant main effects of fructose for leucine, valine, and total BCAA content, though no significant main effects for isoleucine (Figure 6a). Interestingly, after normalizing to nuclei content, a significant interaction effect was observed for each metabolite, during which the cotreatment of cells with fructose and insulin resistance increased each metabolite (Figure 6b). Significant group differences were observed for total BCAA content as well as both isoleucine and valine between insulin-resistant cells and insulin-resistant cells cotreated with fructose (Figure 6b).

## 4. Discussion

Fructose is a common constituent of the Western diet that has been linked with the onset of several metabolic pathologies, including insulin resistance. Collectively, high fructose consumption has previously been associated with several metabolic pathologies due to its high lipogenic potential (details of which are reviewed elsewhere [1,2,3,4]). Additionally, emerging data suggest that despite dramatically lower circulating levels than glucose, circulating fructose is still a correlate and strong predictor of insulin resistance [16]. Previous metabolic assessments of fructose on muscle mitochondrial function and antioxidant capacity in L6 myotubes showed that fructose reduced mitochondrial function following 48 but not 24 h [20]. The same report found fructose decreased AMPK phosphorylation after 48 h of treatment but not sooner [20]. These data suggest fructose down-regulates muscle metabolism; however, an important limitation of this report was the use of 15 mM fructose, which is not physiologically attainable. Conversely, we assessed the effect of a physiologically attainable level of fructose as seen during insulin resistance in humans and found fructose decreases mitochondrial metabolism, which is in line with observations by Jaiswal et al. [20].

In addition to observations linking fructose to reduced mitochondrial metabolism [20,21], David et al. examined the effect of the fructose-induced model of insulin resistance in rats and observed increased circulating BCAA, which is now a commonly observed characteristic of insulin resistance [17]. Interestingly, David et al. observed increased pBCKDHA expression in skeletal muscle (but not other tissues), which is suggestive of reduced BCAA catabolism [17]. Similar to David et al., others have demonstrated rats given a high-fructose diet exhibit increased circulating BCAA and reduced BCAA catabolic capacity in skeletal muscle [18]. Although skeletal muscle is the predominant tissue of BCAA metabolism, the report raises the question of whether increased levels of circulating fructose could depress BCAA catabolism in peripheral tissues. For this reason, the relationship between fructose and BCAA has previously been examined in hepatic tissue. For example, White et al. demonstrated that fructose refeeding can increase the abundance of BCKDK and reduce the amount of PPM1K, thereby reducing BCAA catabolism in the liver [5]. Mechanistically, White et al. proposed that ChREBP activation by excess fructose may promote lipogenesis while simultaneously decreasing the activity of BCAA catabolic enzymes via increased BCKDK:PPM1K [5]. Interestingly, mice given a high-fat, high-fructose diet displayed altered expression of hepatic lipogenic signaling and increased PPM1K but not altered expression or activity of BCKDH [30]. Another interesting finding was that the addition of BCAA to the high-fat, high-fructose diet increased circulating BCAA but did not worsen insulin resistance.

It is known that hepatic fructose clearance is highly efficient and that circulating fructose levels are only a fraction of that of glucose. However, it has also been shown that insulin resistance can be accompanied by increased circulating fructose levels as well (approximating 100 µM) [16]. Collectively, these observations led us to assess the effect of elevated physiological levels of fructose on indicators of BCAA catabolism in a myotube model of insulin resistance. While we observed subtle but significant reductions in mitochondrial and glycolytic metabolism, we observed no effect of fructose on insulin sensitivity or metabolic gene/protein expression related to mitochondrial metabolism, glycolytic metabolism, lipogenesis or BCAA metabolism (though an interaction effect was observed for total BCKDHa expression (Figure 5b)). Despite largely unaltered indicators of BCAA catabolic enzyme expression, we observed elevated extracellular BCAA in fructose-treated cells with concurrent insulin resistance, which is partially in line with observations by David et al. [17]. Thus, it could be that the fructose stimulation in our study was too brief to alter the molecular machinery in a detectable way; however, reduced BCAA metabolism was still occurring. Importantly, it has been shown that the metabolism of BCAA is not only dependent on enzyme activation but also substrate availability, including vitamin cofactors and other contributors [31].

An example was shown following BT2 treatment (a BCKDK inhibitor) of mice, which reduced BCKDH complex phosphorylation in multiple tissues yet only enhanced BCAA metabolism in skeletal muscle [32]. It is noteworthy that fructose treatment in our study did not alter ChREBP expression, which has been directly implicated in down-regulating BCAA catabolism in the liver [5] and may, in part, be how fructose alters BCAA catabolic activity in skeletal muscle [17]. Another important consideration is that the rodent experiments that noted altered BCAA utilization following high-fructose consumption did not assess plasma fructose [17,18], and therefore, it is possible that the levels of fructose at the periphery were higher than those used in our experiments (which were based on observations in humans). However, previous observations in mice indicate that 20% fructose feeding results in similar peripheral blood fructose levels to those used in the present report [27].

Another interesting aspect of the interplay between fructose, insulin resistance, and BCAA may include the abundance of BCAA available in the diet. Several reports have shown that increased BCAA, along with existing pathology, appears to worsen several aspects of metabolic health [11,33,34]. These findings appear to also translate to the myotube in vitro model [35]. Additionally, BCAA restriction appears to improve adiposity during high-carbohydrate feeding [36] and improve aspects of insulin resistance following high-fat feeding [37]. That said, opposing findings have also shown that BCAA-rich whey protein may provide a protective effect against insulin resistance under similar circumstances [38]. Collectively, the interplay between BCAA and other metabolites is complex (and reviewed elsewhere [8,12]); however, as there appear to be interactions between BCAA at varied levels and other substrates, a potential limitation of the present work was the use of only one level of BCAA.

Additionally, another limitation of our study was the use of only a single dose of fructose, though 100 µM was chosen as a relevant reference concentration to observations of those within insulin resistance [16]. Furthermore, our study only assessed response to fructose at a single time point; thus, it is conceivable that longer durations would have had a more pronounced effect on some of the assessed outcomes. We also did not exclude the possibility that fructose altered cell viability; however, it is also important to note that past experiments have used far higher concentrations (25 mM) for longer durations (48 h) in the same cell model [19]. Importantly, for experiments where differences in cell abundance could be influential, we also normalized data to relative nuclei abundance. These limitations aside, our experiments provide foundational information into the effect of physiological fructose levels on muscle metabolism in vitro with and without insulin resistance during hyperglycemic conditions. While our observations suggest fructose may increase extracellular BCAA concentrations during insulin resistance, additional experiments will be necessary to determine if fructose (a) acts directly to alter BCAA metabolism in skeletal muscle leading to an increase in circulating BCAA, (b) increases circulating BCAA by disrupting BCAA metabolism in other tissues (namely liver), (c) acts by promoting systemic insulin resistance which in turn leads to metabolic dysregulation in tissues such as muscle, or (d) a combination thereof. Thus, tissue-specific tracer experiments may be required in order to elucidate the exact mechanisms of reduced BCAA utilization.

## 5. Conclusions

Collectively, our observations demonstrate that fructose may increase extracellular BCAA concentrations independent of the significant reductions in BCAA catabolic machinery. This reduction in BCAA utilization was also parallelled by reduced mitochondrial function, which also occurred independently of changes in related targets at the expressional level. Our results also implicate insulin resistance as a necessary co-contributor in the reduced utilization of myotube BCAA.

## Data Availability

The data that support the findings of this study are presented within the manuscript or supplemental materials and further details are available from the corresponding author upon reasonable request.

## Supplementary Materials

The following supporting information can be downloaded at: https://www.mdpi.com/article/10.3390/nu16111582/s1, Figure S1: Effect of fructose with and without insulin resistance on myotube qRT-PCR loading control; Figure S2: Effect of fructose with and without insulin resistance on myotube Western blot loading control; Figure S3: Effect of fructose with and without insulin resistance on myotube nuclei content; Table S1: qRT-PCR primers; Table S2: Primary antibodies used for western blot experiments; Table S3: Chemicals and experimental reagents.
