# Supplementary material for: Fructose Reduces Mitochondrial Metabolism and Increases Extracellular BCAA during Insulin Resistance in C2C12 Myotubes
Source: Nutrients. 2024 May 23;16(11):1582. doi: 10.3390/nu16111582 (PMC11174010; doi:10.3390/nu16111582)
Supplement: Supplementary file 1 [file nutrients-16-01582-s001.zip › nutrients-3000800-supplementary.pdf]

Supplemental Tables and Legends

Table s1. qRT-PCR primers. Abbreviations: ATP synthase (*Atp5o*), branched-chain aminotransferase 2 (*Bcat2*), branched-chain alpha-keto acid dehydrogenase (*Bckdha*), CCAAT/enhancer-binding protein alpha (*Cebpa*), cytochrome C oxidase Subunit 5A (*Cox5a*), glucose transporter 4 (*Slc2a4* or *Glut4*), hydroxyisobutyrate dehydrogenase (*Hibadh*), lactate dehydrogenase a (*Ldha*), lactate dehydrogenase b (*Ldhb*), carbohydrate response element binding protein as MLX interacting protein-like (*Mlxipl*),nuclear respiratory factor 1 (*Nrf1*), pyruvate dehydrogenase (*Pdh*), peroxisome proliferator-activated receptor gamma coactivator 1 alpha (*Ppargc1a*), peroxisome proliferator-activated receptor gamma (*Pparg*), sterol regulatory element-binding protein (*Srebp1*), Tata box binding protein (*Tbp*), and mitochondrial transcription factor A (*Tfam*).

Gene Abbreviation	**Forward Sequence**	**Reverse Sequence**
Atp5o	5’-AGGCCCTTTGCCAAGCTT-3'	5’-TTCTCCTTAGATGCAGCAGAGTACA-3'
Bcat2	5’-CGGACCCTTCATTCGTCAGA-3'	5’-CCATAGTTCCCCCCCAACTT-3'
Bckdha	5’-CCAGGGTTGGTGGGATGAG-3'	5’-GGCTTCCATGACCTTCTTTCG-3'
Cebp	5′-GTGTGCACGTCTATGCTAAACCA-3’	5′-GCCGTTAGTGAAGAGTCTCAGTTTG-3’
Cox5a	5’-GCTGCATCTGTGAAGAGGACAAC-3'	5’-CAGCTTGTAATGGGTTCCACAGT-3'
Slc2a4(Glut4)	5’-GATGAGAAACGGAAGTTGGAGAGA-3'	5’-GCACCACTGCGATGATCAGA-3'
Hibadh	5’- GCAGCGGTGTGTTCTAGGTC-3’	5’- ACACGTCATAGAGGATGAGTGG-3’
Ldha	5′-GGCTTGTGCCATCAGTATCT-3’	5′-CCCGCCTAAGGTTCTTCATTAT-3′
Ldhb	5’-AGTCTCCCGTGCATCCTCAA-3'	5’-AGGGTGTCCGCACTCTTCCT-3'
Nrf1	5’-ACCCTCAGTCTCACGACTAT-3'	5’-GAACACTCCTCAGACCCTTAAC-3'
Mlxipl	5′-CACTCAGGGAATACACGCCTAC-3′	5′-ATCTTGGTCTTAGGGTCTTCAGG-3′
Pdh	5’-GAAGGCCCTGCATTCAACTTC-3'	5’-ATAGGGACATCAGCACCAGTGA-3'
Ppargc1a	5’-GACAATCCCGAAGACACTACAG-3'	5’-AGAGAGGAGAGAGAGAGAGAGA-3’
Pparg	5′-TTCAGCTCTGGGATGACCTT-3’	5′-CGAAGTTGGTGGGCCAGAAT-3’
Srebp1	5′-ATCGCAAACAAGCTGACCTG-3’	5′-AGATCCAGGTTTGAGGTGGG-3’
Tbp	5’-GGGATTCAGGAAGACCACATA-3’	5’-CCTCACCAACTGTACCATCAG-3’
Tfam	5’-GAAGGGAATGGGAAAGGTAGAG-3'	5’-ACAGGACATGGAAAGCAGATTA-3'

Table s2 Primary antibodies used for western blot experiments. Abbreviations: branched-chain aminotransferase 2 (BCAT2), branched-chain alpha-keto acid dehydrogenase E1α (BCKDHE1α), carbohydrate response element binding protein (ChREBP), fatty acid synthase (FAS), goat polyclonal (GP), horse radish peroxidase conjugate (HRP), mouse monoclonal (MM), oxidative phosphorylation (OXPHOS), rabbit polyclonal (RP), and Sterol regulatory-element binding protein 1 c (SREBP1c). *Notes: Target molecular weight was based on product datasheet. Molecular weights for all targets were verified against sizes suggested by product brochures. * Indicates band is observed at 68 kd.† indicates this molecular weight corresponds to mature SREBP1.*

Antibody Target	*Type*	*Dilution*	*Company*	*Item*	*Approx. Mol Wt.*	*Product Link*
pAkt (Ser 473)	RP	1:1000	SC Biotechnology	sc-7985-R	62kd	p-Akt1/2/3 (Ser 473)
Akt	RP	1:1000	Cell Signaling	9272	62kd	Akt Antibody#9272
β-Actin	MM	1:500	SC Biotechnology	sc-47778	43kd	Datasheet
BCAT2	RP	1:1000	Bioss	BS-6589R	44kd	Datasheet
pBCKDHa(Ser293)	RP	1:1000	AbCam	ab200577	50kd	Phospho BCKDHA (S293)
BCKDHa	RP	1:1000	ProSci	31-325	50kd	BCKDHA Antibody
ChREBP	GP	1:1000	SC Biotechnology	sc-21189	62/78/91/93 kd	ChREBP (P-13): sc-21189
FAS	RP	1:1000	SC Biotechnology	sc-20140	270kd	Fatty Acid Synthase
HIBADH	MM	1:1000	SC Biotechnology	Sc-398288	35kd	Datasheets
PPARγ	RP	1:1000	SC Biotechnology	sc-7196	57kd*	PPARγ (H-100): sc-7196
SREBP1c	RP	1:1000	SC Biotechnology	sc-366	68kd†	SREBP-1 (C-20): sc-366
Total OXPHOS	MM	1:1000	AbCam	ab110413	Varied	Total OXPHOS
Goat Anti-Rabbit IgG	HRP	1:5000	AbCam	ab6721	NA	ab6721
Rabbit Anti-Mouse IgG	HRP	1:5000	AbCam	ab6728	NA	ab6728

Table s3 General chemicals and reagents used in experiments.

EXPERIMENT	DESCRIPTION	VENDOR	PRODUCT #
**CELL CULTURE**	D-Fructose	Sigma	F0127-100G
**CELL CULTURE**	Insulin	Sigma	I6634-50MG
**CELL CULTURE**	DMEM Cell Culture Media	Lonza	95042-512
**CELL CULTURE**	Avantor® Seradigm, Select Grade Fetal Bovine Serum (FBS)	Avantor	1300-500
**CELL CULTURE**	Donor Horse Serum	Corning	35-030-CV
**CELL CULTURE**	Penicillin : Streptomycin 100X	Avantor	SV30010
**CELL CULTURE**	Eiosin Y	Sigma	E4009-5G
**SEAHORSE ASSAY**	Seahorse XFe96/XF Pro FluxPak	Agilent	103792-100
**SEAHORSE ASSAY**	FCCP	Enzo Life Sciences	BML-CM120-0010
**SEAHORSE ASSAY**	Rotenone	Enzo Life Sciences	350-360-G001
**SEAHORSE ASSAY**	Oligomycin	Enzo Life Sciences	380-037-M010
**SEAHORSE ASSAY**	D-Glucose	Sigma	G8769-100ML
**SEAHORSE ASSAY**	Sodium Pyruvate	Sigma	S8636-100ML
**SEAHORSE ASSAY**	L(+)-Glutamine 200 mM 100X	VWR	VWRL0131-0100
**FLUORESCENT STAINING**	DAPI	Biotium	40009
**FLUORESCENT STAINING**	NONYL ACRIDINE ORANGE	Biotium	70012
**FLUORESCENT STAINING**	Nile Red	TCI America	N0659-500MG
**QRT-PCR**	iScript™ cDNA Synthesis Kit	Biorad	1708891
**QRT-PCR**	iQ™ SYBR® Green Supermix	Biorad	1708886
**QRT-PCR**	Microseal 'B' PCR Plate Sealing Film	Biorad	MSB1001
**QRT-PCR**	Hard-Shell® 96-Well PCR Plates,	Biorad	HSP9601
**WESTERN BLOT**	BUFFER RIPA LYSIS/EXTRACTION 100ML	G-Bioscience	786-489
**WESTERN BLOT**	Protease Inhibitor Cocktail	Avantor	M306-5ML
**WESTERN BLOT**	Precision Plus Protein Dual Color Standards	Biorad	1610374
**WESTERN BLOT**	Clarity Western ECL Substrate	Biorad	1705061

Supplemental Figure

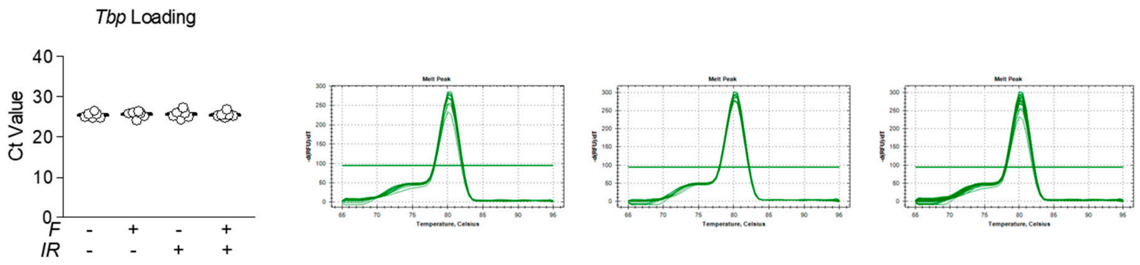

Figure s1 Effect of fructose with and without insulin resistance on myotube qRT-PCR loading control. Effect of fructose at 100µM for 24 hours with and without insulin resistance (IR) on mRNA expression of Tata binding protein (*Tbp*) with melt curves from two independent experiments (left and middle melt curves), and combined (right melt curve).

Notes: Data were analyzed using two-way ANOVA followed by one-way ANOVA with Bonferroni's correction for multiple comparisons used to assess differences in gene expression, protein expression, and lipid content. No statistical differences were observed. Gene expression was conducted using 3 replicates per group across 2 independent cell culture experiments with n=6 for the final analysis.

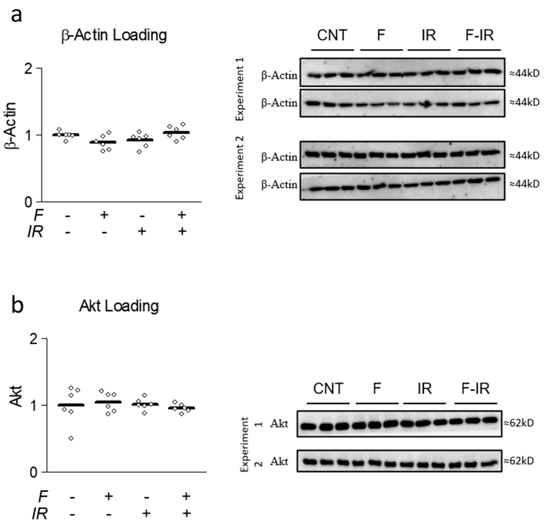

Figure s2 Effect of fructose with and without insulin resistance on myotube Western blot loading control. (a and b) Effect of fructose at 100µM for 24 hours with and without insulin resistance (IR) on protein expression of (a) β-actin or (b) Akt.

Notes: Data were analyzed using two-way ANOVA followed by one-way ANOVA with Bonferroni's correction for multiple comparisons used to assess differences in protein expression. No statistical differences were observed. Protein expression was conducted using 3 replicates per group across 2 independent cell culture experiments with n=6 for the final analysis.

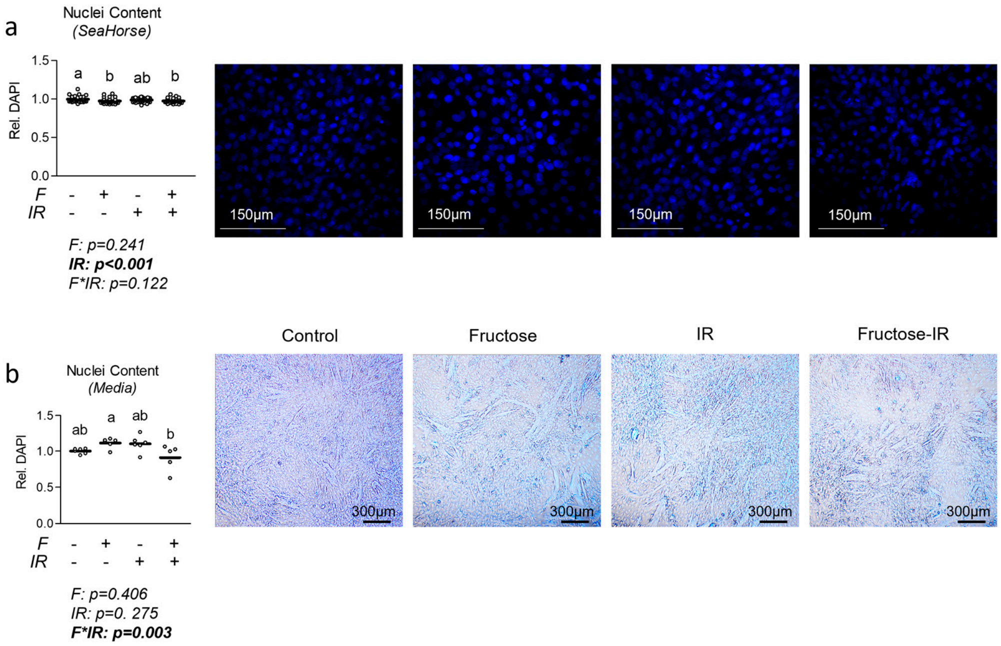

Figure s3 Effect of fructose with and without insulin resistance on myotube nuclei content. (a and b) Effect of fructose at 100µM for 24 hours with and without insulin resistance (IR) on myotube nuclei content from (a) Seahorse assays or (b) media collection experiments.

Notes: Data were analyzed using two-way ANOVA followed by one-way ANOVA with Bonferroni's correction for multiple comparisons used to assess differences in protein expression. Groups with dissimilar letters indicate $p<0.05$ between groups. Nuclei content measurements for Seahorse assays were performed using n=23 individual replicates per treatment condition and were repeated across 2 independent cell culture experiments with n=46 per group in the final analyses. Nuclei content measurements for media collection were performed using n=3 individual replicates per treatment condition and were repeated across 2 independent cell culture experiments with n=5-6 per group in the final analyses.
